# Circulating NAD+ Metabolism-Derived Genes Unveils Prognostic and Peripheral Immune Infiltration in Amyotrophic Lateral Sclerosis

**DOI:** 10.3389/fcell.2022.831273

**Published:** 2022-01-28

**Authors:** Cheng Li, Yu Zhu, Wenzhi Chen, Menghua Li, Mi Yang, Ziyang Shen, Yiyi Zhou, Lulu Wang, Huan Wang, Shu Li, Jiacheng Ma, Mengni Gong, Renshi Xu

**Affiliations:** ^1^ Department of Neurology, Jiangxi Provincial People’s Hospital, Affiliated People’s Hospital of Nanchang University, Nanchang, China; ^2^ Department of Neurology, First Affiliated Hospital of Nanchang University, Nanchang, China; ^3^ Department of Medical Service, The First Hospital of Nanchang, Affiliated Nanchang Hospital of Sun Yat-sen University, Nanchang, China; ^4^ School of Computer and Information Engineering, Jiangxi Agricultural University, Nanchang, China; ^5^ School of Aircraft Engineering, Nanchang Hangkong University, Nanchang, China; ^6^ Medical Examination Center, First Affiliated Hospital of Nanchang University, Nanchang, China

**Keywords:** NAD+, amyotrophic lateral sclerosis, whole blood, peripheral immune infiltration, prognosis

## Abstract

**Background:** Nicotinamide adenine dinucleotide (NAD+) metabolism has drawn more attention on neurodegeneration research; however, the role in Amyotrophic Lateral Sclerosis (ALS) remains to be fully elucidated. Here, the purpose of this study was to investigate whether the circulating NAD+ metabolic-related gene signature could be identified as a reliable biomarker for ALS survival.

**Methods:** A retrospective analysis of whole blood transcriptional profiles and clinical characteristics of 454 ALS patients was conducted in this study. A series of bioinformatics and machine-learning methods were combined to establish NAD+ metabolic-derived risk score (NPRS) to predict overall survival for ALS patients. The associations of clinical characteristic with NPRS were analyzed and compared. Receiver operating characteristic (ROC) and the calibration curve were utilized to assess the efficacy of prognostic model. Besides, the peripheral immune cell infiltration was assessed in different risk subgroups by applying the CIBERSORT algorithm.

**Results:** Abnormal activation of the NAD+ metabolic pathway occurs in the peripheral blood of ALS patients. Four subtypes with distinct prognosis were constructed based on NAD+ metabolism-related gene expression patterns by using the consensus clustering method. A comparison of the expression profiles of genes related to NAD+ metabolism in different subtypes revealed that the synthase of NAD+ was closely associated with prognosis. Seventeen genes were selected to construct prognostic risk signature by LASSO regression. The NPRS exhibited stronger prognostic capacity compared to traditional clinic-pathological parameters. High NPRS was characterized by NAD+ metabolic exuberant with an unfavorable prognosis. The infiltration levels of several immune cells, such as CD4 naive T cells, CD8 T cells, neutrophils and macrophages, are significantly associated with NPRS. Further clinicopathological analysis revealed that NPRS is more appropriate for predicting the prognostic risk of patients with spinal onset. A prognostic nomogram exhibited more accurate survival prediction compared with other clinicopathological features.

**Conclusions:** In conclusion, it was first proposed that the circulating NAD+ metabolism-derived gene signature is a promising biomarker to predict clinical outcomes, and ultimately facilitating the precise management of patients with ALS.

## Introduction

Amyotrophic lateral sclerosis (ALS) is a fatal neurodegenerative disease involving both upper and lower motor neurons that leads to progressive muscle atrophy and motor deficit. The average disease duration of ALS is about 3–5 years (Ludolph et al., 2012; Brown and Al-Chalabi, 2017). The clinical feature of ALS is heterogeneous regarding age and site of disease onset, sex, rate of disease progression, and survival (Xu and Yuan, 2021). There are no effective treatments available in ALS to date; however, the most recently approved edaravone is an antioxidant that acts as a free radical scavenger (Jackson et al., 2019; Petrov et al., 2017). The poor diagnostic and therapeutic approach to ALS may be due to the inability to dynamically evaluate lesions in the central nervous system. Therefore, it is critical to identify biomarkers of clinical progression for ALS, which could be used to monitor and target potential disease-modifying treatments before any irreversible neurodegeneration occurs.

The oxidative stress associated with mitochondrial homeostasis dysfunction and electron transport chain impairment in ALS is a factor that contributes to neurodegeneration (Carrì et al., 2015; Jackson et al., 2019; Smith et al., 2019; Zhu et al., 2020). Nicotinamide adenine dinucleotide (NAD+) is one of the most important coenzymes for redox reactions, as well as being central to energy metabolism. It is also a crucial cofactor for non-redox NAD+ -dependent enzymes, including sirtuins and poly (ADP-ribose) polymerases (PARPs) (Covarrubias et al., 2021). However, low NAD+ levels have been associated with a variety of disorders, including metabolic and neurodegenerative diseases (Xie et al., 2020; Covarrubias et al., 2021). Currently, scientific research suggests that recovery and increasing NAD+ levels may prevent progressive neurodegeneration (Sasaki et al., 2006; Zhang et al., 2016), promote the activity of mitochondrial function (Bonkowski and Sinclair, 2016), reverse the energy metabolism damage and enhance the protection against oxidative stress (Brown et al., 2014; Verdin, 2015).

Nicotinate and nicotinamide metabolism is involving in the biosynthesis and/or degradation pathways for NAD+(Xie et al., 2020). Moreover, the molecules or compounds of nicotinate metabolism are responsible for the *de novo* and Preiss-Handler synthetic pathway, while nicotinamide metabolism relates to the salvage synthetic pathway and consumptions of NAD+. Our previous research demonstrated that the repletion of nicotinamide riboside, a precursor of NAD+, activates mitochondrial unfolded protein response signaling and modulates mitochondrial proteostasis in the brain of SOD1G93A mice (Zhou et al., 2020). Furthermore, studies have found that ALS is associated with impairment of the *de novo* synthetic pathway in NAD+ metabolism, including higher levels of cerebrospinal fluid (CSF) and serum tryptophan, kynurenine, and quinolinic acid, and reduced serum picolinic acid levels (Chen et al., 2010; Lautrup et al., 2019). Compared to household controls, nicotinamide is decreased in both CSF and serum from ALS patients (Blacher et al., 2019). Parallel to the impaired *de novo* pathway, ALS also has a decline in NAD+ due to deficiencies in the nicotinamide monophosphoribosyl transferase (NAMPT)-mediated salvage pathway.

Although there is growing evidence that NAD+ metabolism-related molecules or compounds in body fluids, such as blood or CSF, are altered in ALS. However, no studies have been reported on the NAD+ metabolic pathway concerning the prognostic assessment of ALS. Therefore, the purpose of this study was to investigate whether the peripheral NAD+ metabolic pathway could be identified as a prognostic factor for ALS patients. Transcriptional data of whole blood NAD+ metabolism-related genes (NMRGs) from ALS patients were extracted and a series of bioinformatics and machine learning approaches were combined to screen for robust candidate genes, and establish individualized NAD+ metabolism-derived profiles to predict the overall prognostic value of ALS patients. Besides, we obtained a comprehensive understanding of the peripheral immune regulation of NAD+ metabolism based on the prognostic signature. This provides new insights to elucidate the mechanisms of immune regulation in ALS patients.

## Materials and Methods

### Datasets and Samples

**FIGURE 1:**
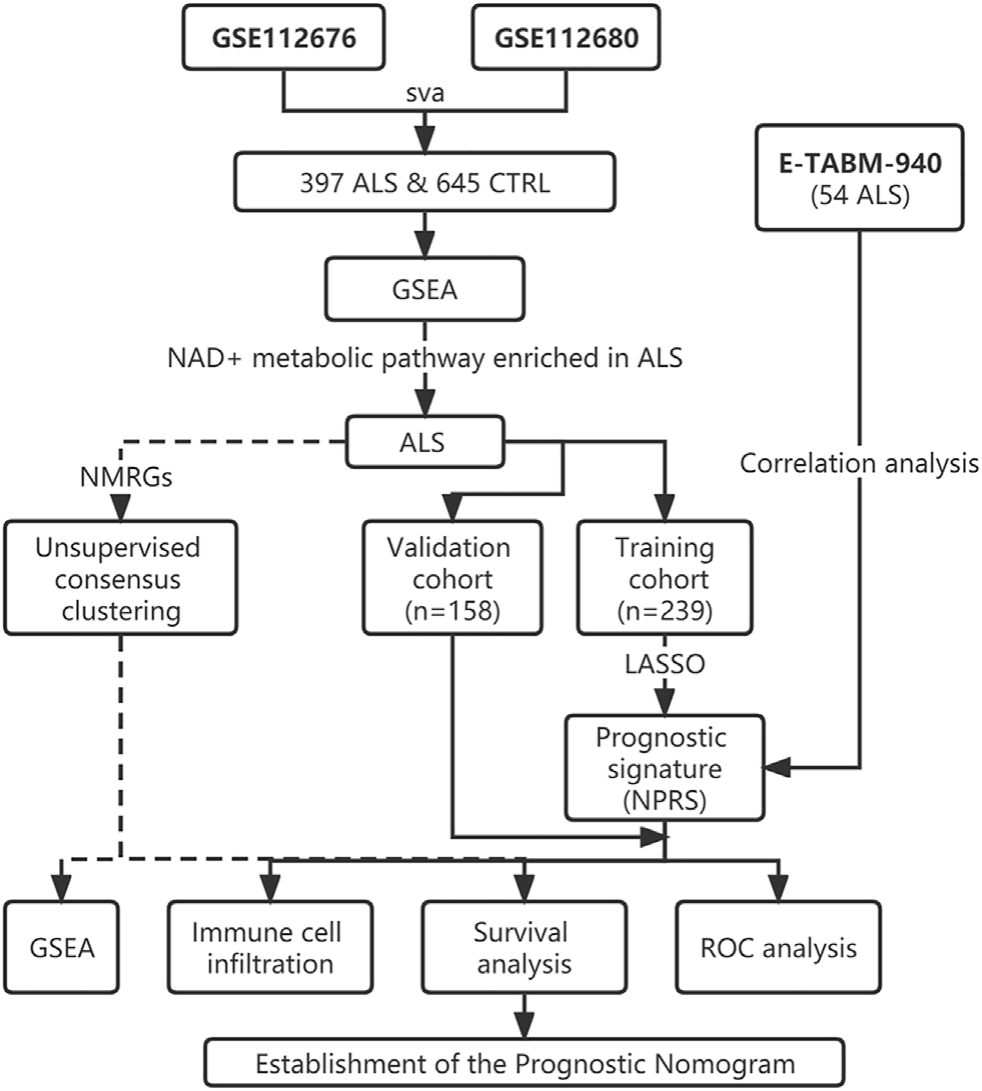
Flow chart of the study design. ALS, Amyotrophic Lateral Sclerosis; CTRL, Control; GSEA, Gene set enrichment analysis; NMRGs, NAD+ metabolism-related genes; NPRS, NAD+ metabolism-related prognostic risk score; ROC, Receptor operating characteristic.

The ALS patient dataset was obtained from the Gene Expression Omnibus (https://www.ncbi.nlm.nih.gov/geo/) and ArrayExpress database (https://www.ebi.ac.uk/arrayexpress/experiments/E-TABM-940/), and the inclusion criteria for the candidate dataset were: ALS, Human gene expression profile, availability of follow-up information (survival information), and related clinical data. GSE112676 and GSE112680, gene expression data were obtained from two microarray platforms (Illumina HumanHT-12 V3.0 and HumanHT-12 V4.0 expression beadchip arrays), were included in our study. GSE112676 (n = 233 ALS and 508 controls) and GSE112680 (n = 164 ALS, 75 mimics, and 137 controls) contained a total of 397 whole blood gene expression data from ALS patients, as described in previous studies (van Rheenen et al., 2018; Swindell et al., 2019). The clinical features of the 397 ALS patients are presented in Table 1. The cases were eventually randomly grouped into a training cohort and a validation cohort for bioinformatics analysis based on the ratio of 6:4. The E-TABM-940 dataset (n = 57 ALS and 23 controls) from ArrayExpress database was used as the external validation cohort (Lincecum et al., 2010). As shown in Figure 1, our study design was briefly described in the flow chart.

**TABLE 1:** Summarization of clinicopathological features of patients in training and validation cohorts.

Variables	—	Training cohortNo. (%)	Validation cohortNo. (%)	*p*-value
No. of patients	—	239	158	—
Sex	Male	144 (60.3)	95 (60.1)	0.98
	Female	95 (39.7)	63 (39.9)	—
Age of onset	—	61.59 (23.12,88.49)	63.05 (26.04,83.93)	0.26
Site of onset	Bulbar	92 (38.5)	54 (34.2)	0.38
	Spinal	147 (61.5)	104 (65.8)	—
Survival time (years)	—	2.95 (0.41,16.51)	2.77 (0.33,13.58)	0.15
Status	Alive	32 (13.4)	23 (14.6)	0.74
	Dead	207 (86.6)	135 (85.4)	—

### Data Processing and Normalization

Raw data of GSE112676 and GSE112680 were downloaded in the supplementary file from the GEO database. We refer to the detailed description of the data processing process and results in the methods and supplementary materials section reported by Swindell et al. (2019) to avoid platform-specific effects and confounding batches. The normal-exponential convolution model was performed for background correction, which using the neqc function (R package: *limma*; function: *neqc*) to normalize and transform the intensities from each sample (Ritchie et al., 2011). The background-corrected GSE112676 and GSE112680 expression matrix were quantile normalized (R package: *limma*; function: *normalizeBetweenArrays*), respectively, and the probes were annotated with gene names using the “illuminaHumanv3.db” package. As reported in the previous study (Swindell et al., 2019), there were large batch effects in the raw analysis of these data. We used the *ComBat* algorithm (R package: *sva*; function: *ComBat*) to remove the apparent batch effect in GSE112676 and GSE112680 according to the method proposed by Swindell et al. (2019). Comparison of the PCA plots before and after normalization and batch corrections from the microarray data showed that the batch effects in the raw data were well removed using the method of Swindell et al. (Supplementary Figure S1). Later, we used the *inner_join* function (R package: *sva*; function: *inner_join*) for two datasets integration and continued to use the *ComBat* function to remove the platform-specific effects. Raw data of E-TABM-940 from the Affymetrix GeneChip Human Genome U133 Plus 2.0 chip platform were pre-processed to an expression matrix using the robust multi-array average method (R package: *affy*; function: *rma*). The probe IDs were annotated with gene names using the annotation file corresponding to the microarray platform, and the expression matrix was quantile normalized for analyses.

NMRGs were obtained from the Kyoto Encyclopedia of Genes and Genomes (KEGG) pathway database (Pathway: hsa00760) and Reactome database (R-HSA-196807). We identified 40 overlapping NMRGs in the GSE112676 and GSE112680 dataset, the expression level of NMRGs was extracted from each case for further analyses.

### Construction and Validation of the NMRGs Signature

Unsupervised consensus clustering analysis was performed prior to the construction of prognostic signature to elucidate the relationship between NAD+ metabolic subtypes and prognosis. Subsequent construction of predictive models based on NMRGs is reasonable only if it is confirmed that NAD+ metabolic subtypes affect the prognosis of ALS patients. In the training cohort, using the Least Absolute Shrinkage And Selection Operator (LASSO) regression with 10-fold cross-validated to screen out NMRGs associated with survival in ALS patients. In LASSO regression, a penalty term is added based on the least square to compress the estimated parameters, select the independent variable that has a great influence on the dependent variable and calculate the corresponding regression coefficient, and finally obtains a simplified model that successfully prevents overfitting. Here, the *glmnet* package was applied to determine the optimal lambda value corresponding to the minimum of the error mean via cross-validation. These NMRGs associated with prognosis (survival status and survival time) in ALS patients screened by LASSO regression were used to construct prognostic risk signature based on regression coefficients. ALS patients were then divided into high-risk or low-risk groups according to the median risk score. The NAD+ metabolism-related prognostic risk score (NPRS) of each sample was calculated using the formula: NPRS = ΣExp (mRNAί) × Coefficient (mRNAί). Kaplan-Meier survival analysis and time-dependent ROC curves were used to evaluate the prognostic predictive performance. Furthermore, survival analysis and time-dependent ROC were also validated in the validation cohort and the external validation cohort (R package: *timeROC*).

The independent predictive variables identified by LASSO regression were used to construct the predicted nomogram and the corresponding calibration curves (R package: *rms*). The concordance index was used to evaluate the accuracy of the predictive performance of the nomogram, and the diagnostic accuracy was measured by ROC curve analysis (R package: *survivalROC*). Univariate and multivariate Cox regression analyses were conducted to profile independent prognostic parameters.

### Investigation of Immune Cell Type Fractions in ALS Blood

Considering that NAD+ metabolism and immunity are closely linked (Navas and Carnero, 2021), and peripheral immune cells are closely associated with disease progression in ALS patients (Murdock et al., 2017). Therefore, it is difficult to dissociate peripheral immune infiltration from the study. Cell-type Identification By Estimating Relative Subsets Of RNA Transcripts (CIBERSORT) (Newman et al., 2015) analysis was employed to estimate the proportions of 22 human immune cell subsets according to the gene expression data. Only samples with *p* < 0.01 in CIBERSORT analysis results were used in the subsequent analysis of differential immune infiltration levels.

### Additional Bioinformatic Analyses

To elucidate the biological phenotype regulated by the NMRGs in the blood of ALS patients, the unsupervised consensus clustering (R package: *ConsensuClusterPlus*) (Wilkerson and Hayes, 2010) was used to obtain clusters based on “pam” method with 1,000 iterations and resample rate of 80%. Principal component analysis (PCA) and uniform manifold approximation and projection for dimension reduction (UMAP) (Trozzi et al., 2021) methods were used to evaluate gene expression patterns in peripheral blood of different subclusters of ALS. The GSEA software version: 4.1.0 (https://www.gsea-msigdb.org/gsea/index.jsp) was used to illustrate the enriched KEGG and Reactome pathways among different ALS subtypes.

### Statistical Analysis

All Statistical analyses and visualization were carried out by R version 4.0.2 (http://www.r-project.org). Student’s t-test and Wilcoxon signed-rank test were used to estimating the differences between the two groups, and the Kruskal–Wallis test was used to compare more than two groups. Kaplan-Meier analyses and log-rank tests were used to assess the survival differences between subtypes of ALS patients. The Spearman analysis computed the correlation coefficients between NPRS and ALS-related parameters. *p* < 0.05 was regarded as statistically significant.

## Results

### The Landscape of NMRGs in the Whole Blood of ALS

**FIGURE 2:**
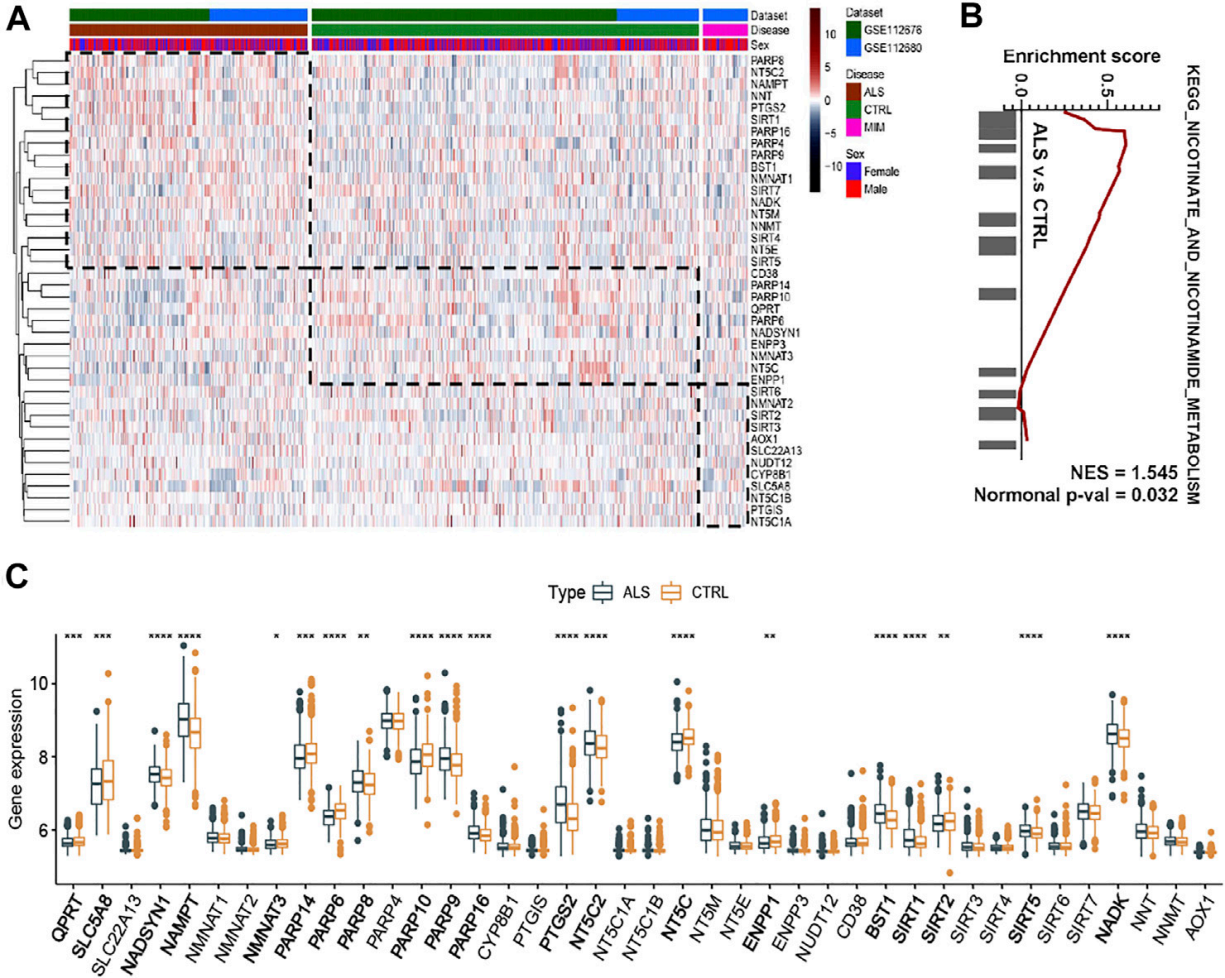
NAD+ metabolism-related molecular patterns and GSEA in whole blood of ALS patients **(A)** Heatmap of NAD+ metabolism-related genes in ALS patients, controls and ALS mimics **(B)** Differential expression of NAD metabolism-related genes in whole blood of ALS and controls **(C)** GSEA showed that the nicotinate and nicotinamide metabolism pathway are differentially enriched in ALS patients. ALS, Amyotrophic Lateral Sclerosis; CTRL, control; MIM, mimic; NES, normalized enrichment score; Normal p-val, normalized *p* value. *p < 0.05, **p < 0.01, ***p < 0.001, and ****p < 0.0001.

Our previous study reported the aberrant expression of NAD+ Metabolism-related coenzymes was identified in the brain of SOD1^G93A^ mice (Zhou et al., 2020). Therefore, we attempted to systematically observe the expression patterns of NMRGs in the whole blood of ALS patients. Based on whole blood transcriptional microarray analysis (from GSE112676 and GSE112680) of 397 ALS patients, 645 controls, and 75 ALS-mimics, significant changes in the transcriptional levels of NMRGs in ALS whole blood were described (Figures 2A,B). The expression of NADSYN1, NAMPT, PARP8/9/16, PTGS2, NT5C2, BST1, SIRT1/5, and NADK was significantly up-regulated in ALS, whereas QPRT, SLC5A8, NMNAT3, PARP6/14/10, NT5C, ENPP1, and SIRT2 were significantly downregulated. GSEA (Figure 2C) was used to identify differences in transcriptional mechanisms between whole blood from ALS patients and controls, and the results revealed that the nicotinate and nicotinamide metabolism pathway (normalized enrichment score [NES] = 1.545, normalized *p* value = 0.032) was significantly enriched in ALS.

### Identification of NAD+ Metabolic Subtype

**FIGURE 3:**
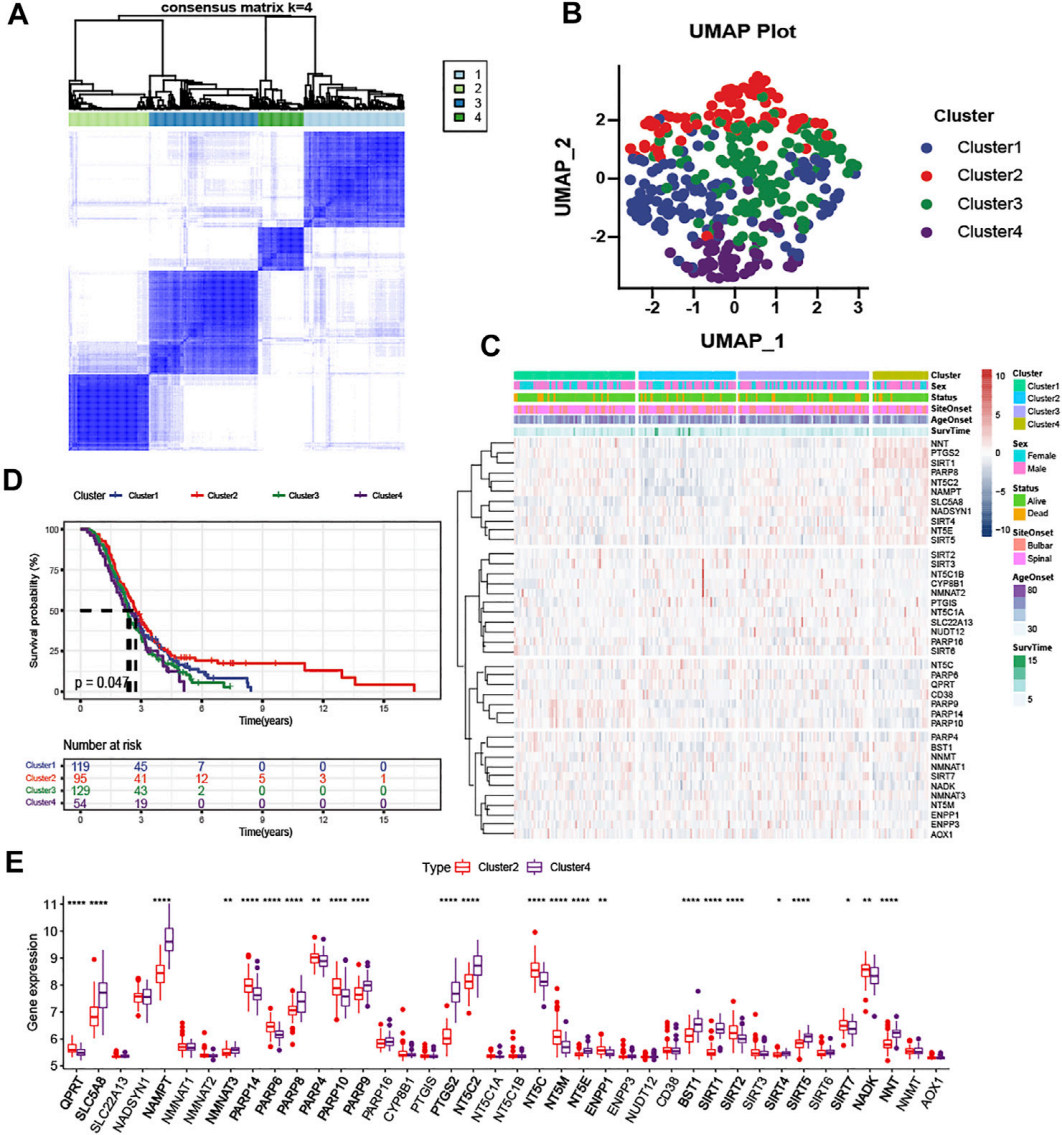
Clustering analysis based on NAD + metabolism-related gene expression profile identifies prognosis of ALS patients **(A)** Consensus clustering matrix for k = 4 **(B)** UMAP analysis confirmed the classification **(C)** Heatmap depicted the expression levels of NAD+ metabolism-related genes among the four subtypes **(D)** Kaplan-Meier survival analysis for patients with ALS in four clusters **(E)** Boxplots showed differential expression of NAD+ metabolism-related genes between Cluster2 and Cluster4. *p < 0.05; **p < 0.01; ***p < 0.001, and ****p < 0.0001.

According to the expression level of NMRGs, the optimal clustering stability of k = 2 to 10 was achieved when k = 4 (Figure 3A and Supplementary Figure S2A,B). The 397 ALS patients were divided into four subtypes: Cluster1 (n = 129), Cluster2 (n = 95), Cluster3 (n = 129), and Cluster4 (n = 54). UMAP and PCA analysis revealed large differences between Cluster2 and the rest of subtypes, particularly with Cluster4 (Figure 3B and Supplementary Figure S2C). The heatmap (Figure 3C) also displayed that the expression patterns of NMRGs were different in Cluster2 and Cluster4. Besides, Kaplan-Meier analysis showed that ALS patients with Cluster2 had the most satisfactory outcome (Figure 3D), while ALS patients with Cluster4 had more rapid disease progression. Compared to Cluster2, eleven genes (NNT, PTGS2, SIRT1, PARP8, NT5C2, NAMPT, SLC5A8, NADSYN1, SIRT4, NT5E, SIRT5) were significantly upregulated in Cluster4, while six genes (NT5C, PARP6, QPRT, CD38, PARP14, PARP10) were significantly downregulated in Cluster4 (Figure 3E).

**FIGURE 4:**
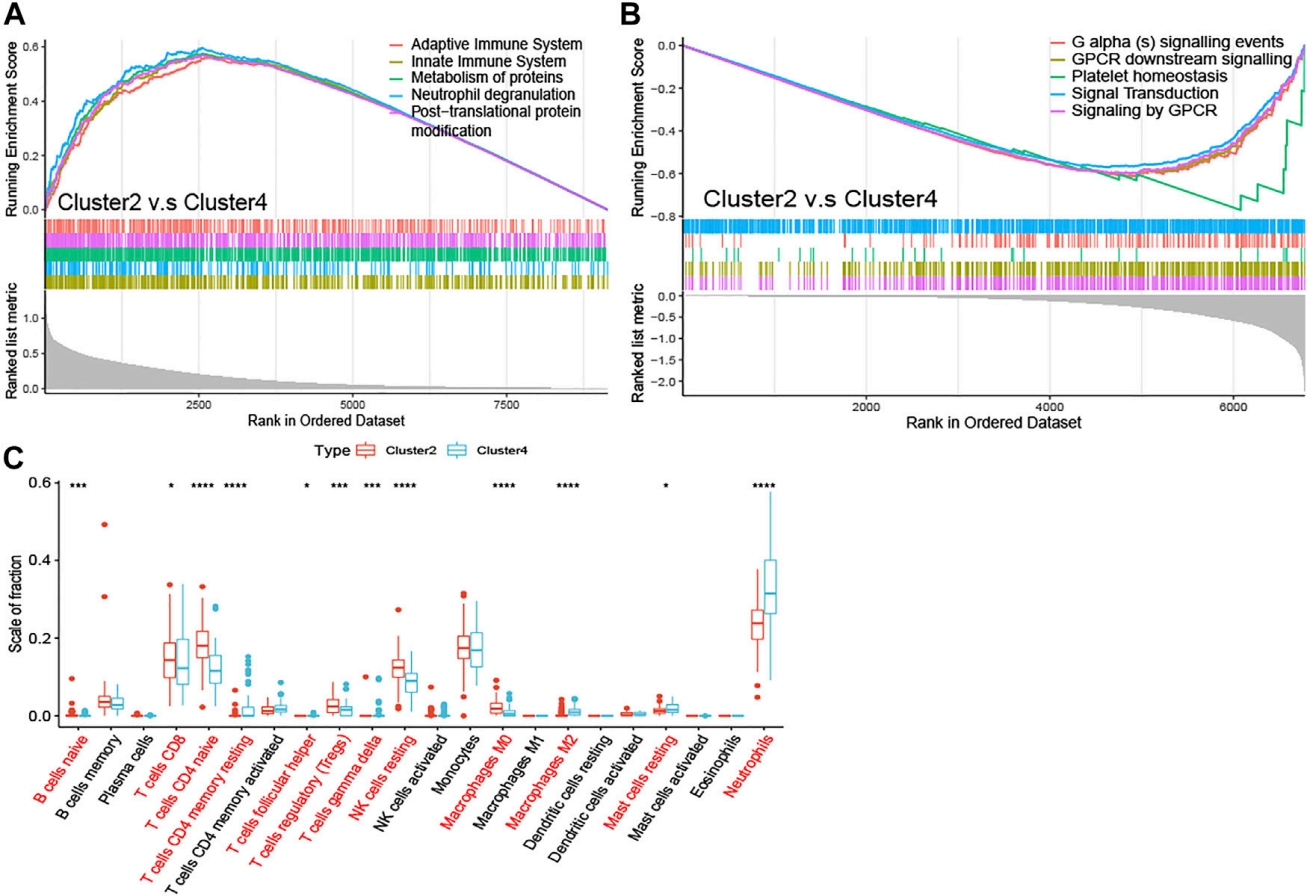
Enrichment analysis and peripheral immune infiltration characteristics of NAD+ metabolic subtypes with distinct prognosis **(A)** GSEA for Cluster2 and Cluster4. Immune pathways and protein metabolic pathways were significantly enriched in Cluster2 **(B)** and GPCR signaling-related pathways were significantly enriched in Cluster4 **(C)** Boxplots showed immune cell infiltration differences between Cluster2 and Cluster4. *p < 0.05; **p < 0.01; ***p < 0.001, and ****p < 0.0001.

To elucidate the potential regulatory mechanisms leading to prognostic differences between Cluster2 and Cluster4 by GSEA. Adaptive immune system, innate immune system, neutrophil degranulation, post-translational protein modification, and metabolism of proteins were predominantly enriched in the Cluster2 (Figure 4A; All *p* < 0.01), which are patients with better survival, wheares G Protein-Coupled Receptors (GPCR) signaling related pathways and platelet homeostasis pathway were significantly enriched in the Cluster4 (Figure 4B; All *p* < 0.01).

To investigate the relationship between subtypes and immune cell infiltration in the whole blood of ALS patients, the CIBERSORT method was used to evaluate the relative immune cell levels of four subtypes (Figure 4C). The B cells, CD8 T cells, CD4 naive T cells, regulatory T cells (Tregs), and natural killer (NK) resting cells were significantly higher infiltration in the blood of patients with Cluster2, while the neutrophils were lower infiltrated. The CD4 memory T cells (both resting and activated), follicular helper T cells (Tfh), gamma delta T cells (Tgd), M2 macrophages, and mast resting cells were significantly increased in patients with Cluster4, while CD4 naive cells and M0 macrophages were significantly reduced.

### Construction of the NAD+ Metabolism-Related Prognostic Risk Score

**FIGURE 5:**
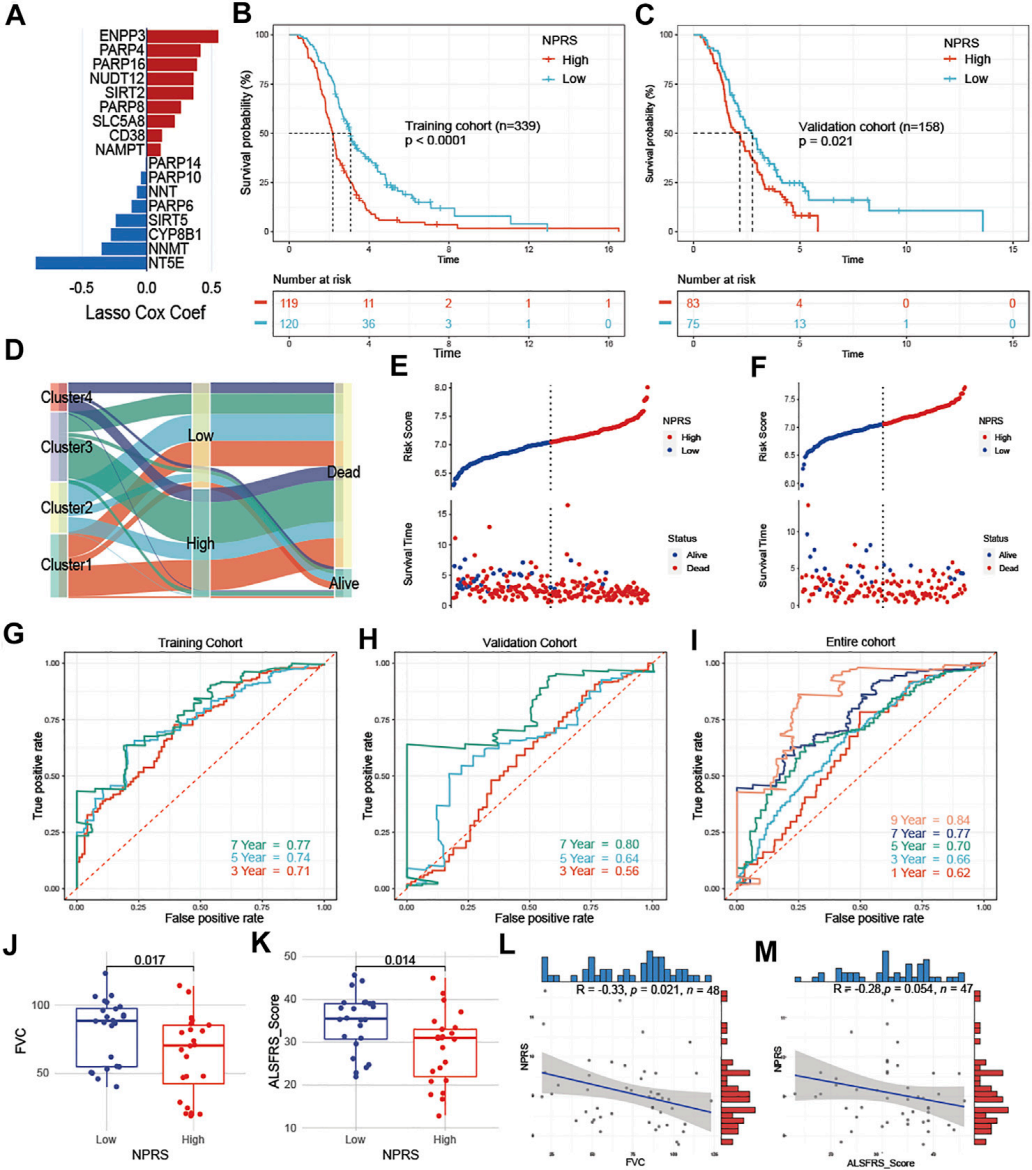
Development and assessment of a prognostic NAD+ metabolism-related signature for ALS patients **(A)** An ensemble of 17 genes remained with individual coefficients **(B)** and **(C)** Kaplan-Meier survival analysis for patients with high- and low- NPRS in the training (B) and validation cohorts (C), ALS patients in the high group had worse prognostic survival than those in the low group **(D)** Sankey plot of four clusters distribution in groups with different NPRS, and survival outcomes **(E)** and **(F)** Risk plots showed that patients with low scores in the training (E) and validation cohorts (F) had better survival **(G–I)** In the training (G), validation (H), and entire (I) cohorts, time-dependent receiver operating characteristic analysis showed NPRS had better performance in predicting the survival of ALS patients **(J)** and **(K)** Patients in the low NPRS group had better FVC (J) and ALSFRS (K) scores compared to the high NPRS group in E-TABM-940 **(L)** and **(M)** In E-TABM-940, NPRS was negatively correlated with FVC (L) or ALSFRS (M) scores, respectively.

ALS patients were randomly divided into a training cohort (n = 239) and a validation cohort (n = 158) at a ratio of 6:4. In the training cohort, we screened the most robust prognostic genes in NMRGs using the LASSO algorithm with an optimal lambda value of 0.0429 (Supplementary Figure S3A,B). Seventeen genes were identified to construct the NMRGs-related prognostic signature, as shown in Figure 5A. The NPRS was calculated for each sample according to the established formula. All ALS patients were separated into high- and low- NPRS groups according to the median value of the NPRS in the training cohort. Kaplan-Meier analysis (Figures 5B,C) demonstrated that ALS patients in the high-NPRS group were more rapidly progressive than those in the low-NPRS group in both the training cohort (*p* < 0.0001) and the validation cohort (*p* = 0.021). Sankey plots (Figure 5D) showed that Cluster2 patients were mostly concentrated in the low-NPRS group, while these subtypes with poorer prognoses were largely distributed in the high-NPRS group. Furthermore, fewer patients in the high-NPRS group were alive compared to the low-NPRS group, which means that more patients were dead in the high-NPRS group. Correspondingly, Cluster2 had the lowest NPRS among the four subtypes (Supplementary Figure S4). The subtype, sex, site of onset, age at onset, survival status, and duration, as well as immune infiltration score under the high- and low- NPRS subgroups are illustrated in the heatmap (Supplementary Figure S5). Risk plots showing the association of NPRS with survival time and status in the training (Figure 5E) and validation (Figure 5F) cohorts, which demonstrated that NPRS has predictive value for prognosis in ALS patients.

To investigate the predictive accuracy of NPRS for prognosis, receiver operating characteristic curve (ROC) analysis was used and the area under the curve (AUC) values at 3, 5, and 7 years were compared. The 3-, 5-, and 7-year AUC values for assessing the predictive accuracy of NPRS in the training cohort were 0.71, 0.74, and 0.77, respectively (Figure 5G); the 3-, 5-, and 7-year AUC values in the validation cohort were 0.56, 0.64, and 0.8, respectively (Figure 5H); and the 1-, 3-, 5-, 7-, and 9-year AUC values in the entire cohort were 0.62, 0.66, 0.7, 0.77, and 0.84, respectively (Figure 5I). Furthermore, the dataset of ALS patients in E-TABM-940 was used as an external cohort to test the predictive value of NPRS. The above formula was first applied to calculate the NPRS for each patient, which was assessed by observing the relationship between NPRS and clinical parameters related to ALS. ALS patients in the external cohort were divided into high and low-risk groups according to the median score, and subgroup analysis showed that both FVC (Figure 5J) and ALSFRS (Figure 5K) were significantly lower in the high NPRS group compared to patients in the low group. Moreover, analysis of correlations identified a significant negative correlation between NPRS and FVC (Figure 5L, r = −0.33 *p* = 0.021); NPRS also showed a negative trend with ALSFRS scores, although it was nearly to reach statistical power (Figure 5M, r = −0.28, *p* = 0.054).

**FIGURE 6:**
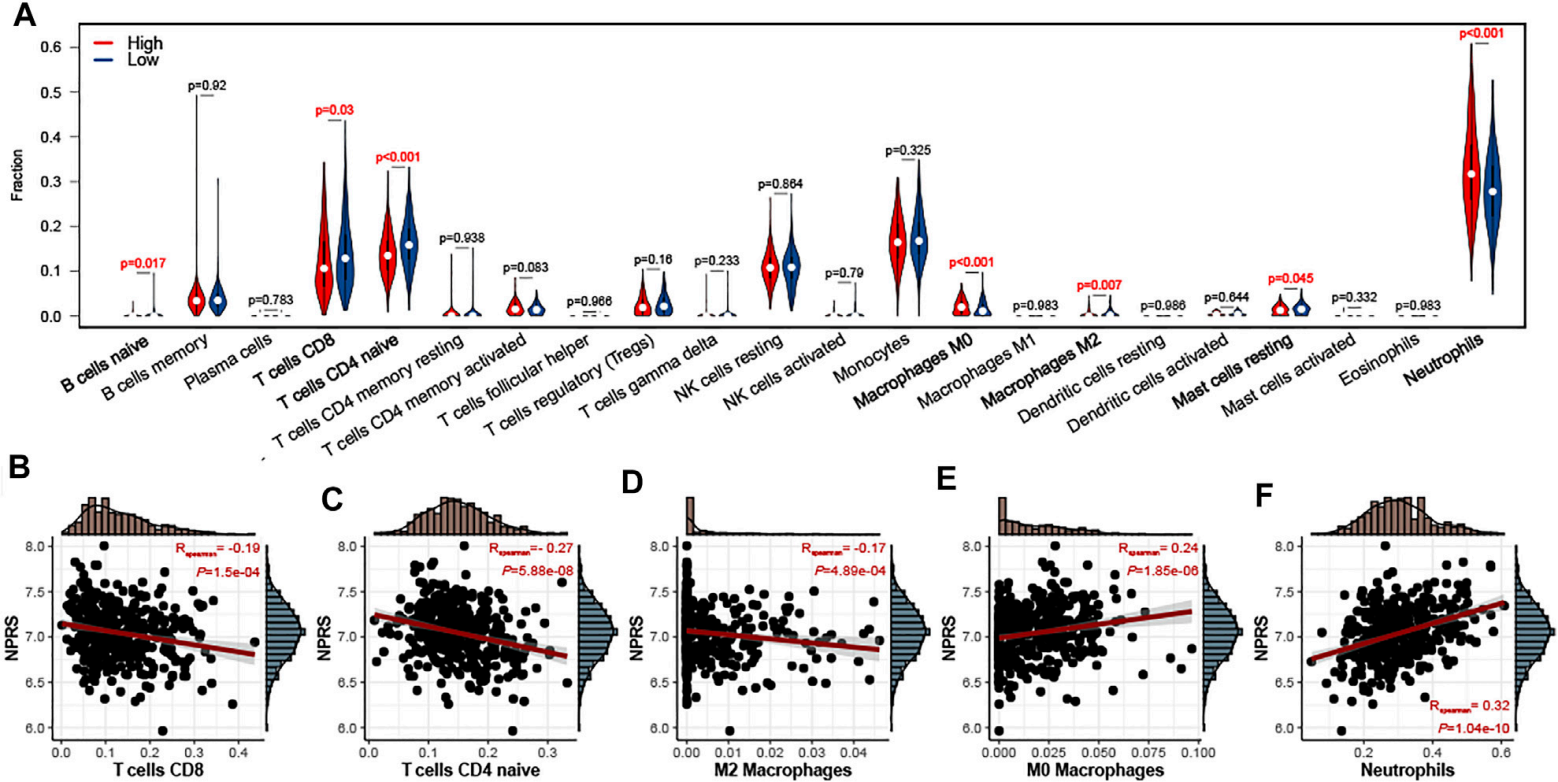
Estimation of peripheral immune cell infiltration by the prognostic signature **(A)** The peripheral infiltrating levels in the high- and low- NPRS groups by using CIBERSORT algorithms. The violin plot showed the differences in peripheral immune infiltration score between the high- and low- NPRS groups **(B-F)** The Spearman correlation between NPRS and the fraction of peripheral immune cells is shown. Scatterplots depicts that NPRS is negatively correlated with CD8 T cells (B), CD4 naive T cells (C), and M2 macrophages (D), while positively correlated with M0 macrophages **(E)** and neutrophils **(F)**.

To evaluate the relationship between NPRS and immune cell infiltration in the whole blood of ALS, the CIBERSORT algorithm was used to analyze immune cell differences in patients (Figure 6A). Patients with higher scores had significantly increased neutrophils and M0 macrophages, while B naive cells, M2 macrophages, mast resting cells, CD8 T cells, and CD4 naive T cells were relatively lower. Analyses of correlations suggested that CD8 T cells (Figure 6B; r = −0.19, *p* < 0.001), CD4 naive T cells (Figure 6C; r = −0.27, *p* < 0.001), and M2 macrophages (Figure 6D; r = −0.17, *p* < 0.001) were negatively correlated with NPRS, while M0 macrophages (Figure 6E; r = −0.24, *p* < 0.001), and neutrophils (Figure 6F; r = −0.32, *p* < 0.001) were positively correlated.

### Clinicopathological Analysis for NPRS

**FIGURE 7:**
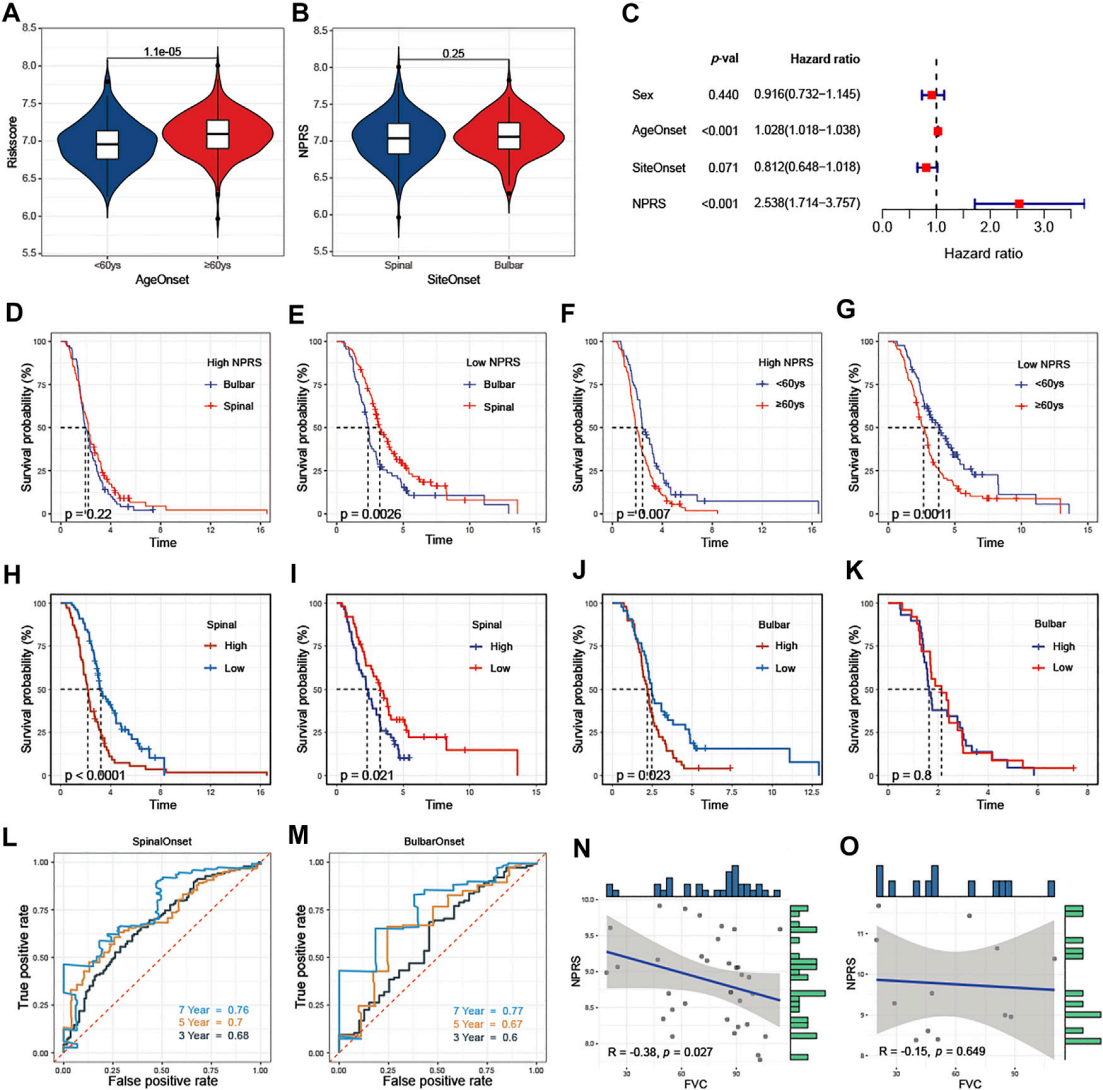
Association of NPRS with clinicopathological features **(A)** NPRS was significantly higher in patients with elderly onset **(B)** There was no difference in NPRS between patients with different sites of onset **(C)** Age at onset and NPRS were independent prognostic risk factors in the multivariate COX regression model **(D)** and **(E)** Progression in patients with high NPRS **(D)** is not affected by site of disease onset, while survival differences are observed in patients with low risk scores **(E) (F)** and **(G)** Differences in survival risk by age of onset are not affected by high- **(F)** or low- **(G)** NPRS patients. Comparison of the prognostic capacities and survival outcomes of the risk score for patients with different features of onset **(H)** and **(I)** Both in training (H) and validation (I) cohorts, high-NPRS patients with spinal onset have poor survival **(J)** and **(K)** In the bulbar onset group, high-NPRS patients showed worsen survival compare to the low-NPRS group in the training cohort **(J)** but without survival differences between high- and low- NPRS patients in the validation cohort (K) **(L)** and **(M)** In the entire cohort, time-dependent receiver operating characteristic analysis of NPRS in predicting the survival of ALS patients with spinal **(L)** or bulbar (M) onset **(N)** and **(O)** In E-TABM-940, negative correlated between NPRS and FVC in ALS patients with spinal onset (N) but not in patients with bulbar onset (O).

Clinicopathological analysis revealed that higher NPRS in patients with age at onset ≥60 years than in patients <60 years (Figure 7A). No statistical difference in NPRS among ALS patients with different sites of onset (Figure 7B). To determine whether the predictive capacity of the prognostic features of signature was independent of other traditional clinical characteristics (including sex, age at onset, and site of onset), we performed univariate (Supplementary Figure S6) and multivariate (Figure 7C) Cox regression analyses of these parameters in the entire cohort. The results confirmed that age at onset (HR = 1.028, 95%CI: 1.018-1.038) and NPRS (HR = 2.538, 95%CI: 1.714-3.757) were independent risk factors for ALS progression. Additionally, we observed that the overall survival of patients with spinal initiation in the low-NPRS groups was superior (Figure 7D); however, the survival benefit of spinal initiation was relatively not significant in patients with high-NPRS (Figure 7E). Furthermore, patients with an age of onset <60 years had a favorable survival advantage over elder patients in both high- (Figure 7F) and low- NPRS groups (Figure 7G).

The prognostic value of NPRS for ALS patients with different sites of onset was investigated. Both in training (Figure 7H) and validation (Figure 7I) cohorts, survival analysis indicated that the prognosis of patients with spinal initiation in the high-NPRS group was significantly worse compared with the low-NPRS group. Rapid progression was observed in high NPRS patients with bulbar onset in the training cohort (Figure 7J), but could not be identified in the validation cohort. (Figure 7K). Furthermore, the 3- and 5-year AUC values for assessing the predictive accuracy of NPRS were higher in patients with spinal onset (Figure 7L; 3-year: 0.68; 5-year: 0.7) than patients with bulbar onset (Figure 7M; 3-year: 0.6; 5-year: 0.67). Moreover, a significant negative correlation between NPRS and FVC scores was also observed in patients with spinal initiation (Figure 7N) in the external cohort, but not in patients with bulbar onset (Figure 7O).

### Establishment of the Prognostic Nomogram

**FIGURE 8:**
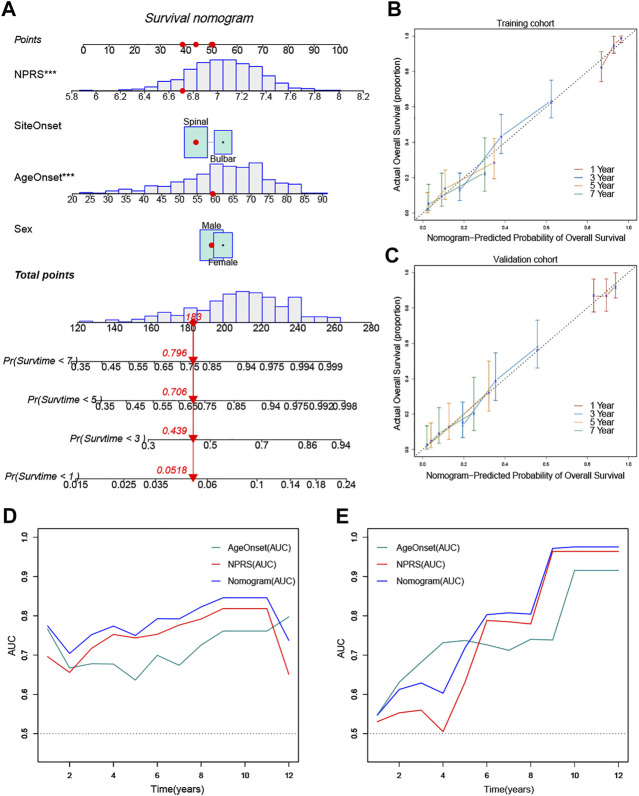
Construction and validation of a predictive nomogram **(A)** The nomogram for predicting the overall survival of patients with ALS at 1, 3, 5, and 7 years **(B)** and **(C)** The nomogram showed high accuracy in the calibration analysis **(D)** and **(E)** The nomogram exhibited the most powerful capacity for survival prediction compared with other clinicopathological features in both training and validation cohorts. ***p < 0.001.

To quantify the prognostic risk assessment of ALS patients, a predictive nomogram was constructed to measure the probability of ALS survival at 1, 3, 5, and 7 years (Figure 8A). In the calibration analysis, the prediction lines of the nomogram for 1-, 3-, 5- and 7-year survival probability were extremely close to the ideal performance, suggesting a great accuracy of the nomogram (Figures 8B,C). Then, we performed ROC analysis to verify the predictive value of the nomogram. The AUCs of nomogram were superior to the single independent predictor in both training and validation cohorts (Figures 8D,E). Therefore, the nomogram provided the best benefit for survival prediction compared to other clinical parameters.

## Discussion

As an important coenzyme for redox reactions in the cytosol and mitochondria, NAD+ is essential for enabling the most basic biological functions of the cell, including the glycolysis, the tricarboxylic acid cycle, and β-oxidation of fatty acids. NAD+ can be synthesized in cells from precursors via three major pathways: the *de novo* pathway, Preiss-Handler pathway, and salvage pathway. The salvage pathway mediated by the NAMPT is required for the majority of NAD+ production in mammalian cells (Evans et al., 2002; Revollo et al., 2004). SIRTs, PARPs, and cyclic ADPR (cADPR) synthetases are involved in the catalytic process of NAD+ consumption. Interestingly, decreased NAD+ levels may be a risk factor for age-related neurodegeneration (Lautrup et al., 2019). Indeed, low levels of NAD+ were assumed to be a consequence of aging (Clement et al., 2019), including in the human brain and CSF(Guest et al., 2014; Zhu et al., 2015), serving as a neuroprotective and anti-inflammatory molecule (Verdin, 2015).

In our previous works, the NAMPT-mediated NAD+ biosynthesis was the main reason for the NAD+ levels decline, the replenishment of intracellular NAD+ by providing NR might modulate the mitochondrial proteostasis and improve the adult neurogenesis through activating the mitochondrial unfolded protein response signaling in the brain of SOD1^G93A^ mice (Zhou et al., 2020). Further, either NAMPT overexpression or NMN supplementation enhances resistance to oxidative stress by increasing mitochondrial and cytoplasmic NAD+ levels *in vitro* models of ALS (Harlan et al., 2016). Notably, key molecules of the tryptophan-nicotinamide metabolic pathway were also significantly altered in serum of ALS patients, and sera levels of nicotinamide correlated with better scores of ALSFRS. Likewise, low nicotinamide levels were observed in the cerebrospinal fluid of ALS patients (Blacher et al., 2019). Alterations in molecules associated with the NAD+ metabolic pathway in peripheral blood are highly suggestive of its potential as a biomarker for ALS. However, this possibility has not been confirmed.

In this study, We collected as much of the transcriptional dataset of peripheral blood from ALS patients with clinical parameters as possible from public databases. Firstly, we explored the aberrant NAD+ metabolic pathway in the blood of ALS patients using GSEA. Then we identified different NAD+ metabolic subtypes based on the expression patterns of NMRGs and compared the prognosis and immune infiltration differences between subtypes. This was aimed to clarify whether NMRGs in the blood of ALS patients could predict prognosis and peripheral immune infiltration. Finally, peripheral blood prognostic signatures of ALS patients were constructed using the LASSO regression method, and subsequent subgroup analysis and construction of a prognostic nomogram were performed.

The expression patterns, prognostic values, and effects on the immune cell infiltration of NMRGs in peripheral blood of ALS were demonstrated. In contrast to the trend towards low expression in the central nervous system of ALS, transcriptional levels of NAMPT were significantly higher in blood compared to the control population. Similarly, elevated trends were found for NADSYN1, an NAD+ synthetic enzyme involved in the final step of the Preiss-Handler pathway (also involved in the *de novo* pathway). However, transcript expression of QPRT, a key enzyme of the tryptophan *de novo* synthesis pathway, was significantly lower in the whole blood of ALS. Regarding changes in NAD+ consuming enzymes, SIRTs and the biosynthetic catalase of NADP+ (NNT and NADK) showed an increasing trend in blood, whereas PARPs were inconsistently differentially expressed in ALS. This suggests that the NAD+ metabolic pathway is activated in the blood of ALS patients, and indirectly confirmed by the GSEA results. The synthesis of NAD+ in the peripheral blood of ALS is predominately mediated by the Preiss-Handler pathway and the salvage pathway. Although metabolites of the *de novo* pathway are increased in the serum of ALS patients (Chen et al., 2010; Blacher et al., 2019), the low expression of QPRT implies that the *de novo* pathway is attenuated in blood.

Moreover, unsupervised consensus clustering analysis of NMRGs expression patterns revealed that the expression of QPRT was relative lower in patients with Cluster4 compared to those with Cluster2. This suggested that the progressive reduction of NAD+ synthesis by the *de novo* pathway in the blood of ALS patients may be related with rapid disease progression. Meanwhile, the Preiss-Handler pathway mediated by SLC5A8 and NMNAT3, and the salvage pathway mediated by NAMPT were relatively up-regulated in patients with Cluster4 compared to those with Cluster2. This result also supports our speculation above that excessive activation of the non-*de novo* synthesis pathway in the blood may indicate a poor prognosis for patients. The expression trends of NAD+ consuming enzymes were inconsistent, which may be related to NAD+ subcellular homeostasis. Indeed, NAD+ is involved in multiple biological processes within a variety of organelles (Xie et al., 2020).

The LASSO regression model was employed to screen for the most robust biomarkers to create an NMRG-related prognostic signature, as well as a risk score constructed based on the signature. Subsequently, the NPRS was validated in different cohorts to have a favorable prognostic value for patients with ALS, in which patients with low-NPRS showed better survival and vice versa. The AUC values of the prognostic signature suggested a greater predictive power beyond 5 years. Furthermore, Cluster2, which had a better prognosis in clusters, possessed a lower NPRS, and patients with elderly onset of ALS had a higher score than youngers. The fact is that age of onset is one of the risk factors influencing the prognosis of ALS patients (Testa et al., 2004). Besides, we also found a better prognostic value of the signature in patients with spinal onset, which may be related to the variation in symptoms due to different sites of onset. ALS with bulbar onset is more likely to cause dietary and ventilatory disorders (Brown and Al-Chalabi, 2017), leading to disturbances in energy metabolism, which increases the difficulty of prediction. A prognostic nomogram was built to quantify risk assessment and survival probability based on NPRS, sex, age and site of onset for ALS patients. Compared to other clinical features, the nomogram demonstrated the highest accuracy and discrimination in survival prediction.

As an essential metabolite for cellular homeostasis, NAD+, and its metabolic enzymes are also important for the regulation of immune responses (Navas and Carnero, 2021). The CIBERSORT algorithm evaluated the immune cell infiltration in ALS whole blood. We identified several peripheral blood immune cells potentially associated with prognosis in ALS, mainly involving B naive cells, CD4 naive T cells, CD8 T cells, M0 and M2 macrophages, and neutrophils. M0 macrophages, and neutrophils were higher infiltrated in patients with worsen survival, while B naive cells, M2 macrophages, resting mast cells, CD4 naive T cells, and CD8 T cells were relatively lower infiltrated. Several immune cells were correlated with NPRS, such as neutrophils and M0 macrophages, which were positively correlated, but CD4 naive cells, CD8 T cells, and M2 macrophages were negatively correlated. This may revealed that neutrophils and M0 macrophages in the blood of ALS patients may promote disease progression, while CD4 naive T cells and CD8 T cells may act as retarders. Recent studies have reported that increased neutrophils and monocytes in peripheral immunity in ALS patients (Murdock et al., 2016,^2017^; Gustafson et al., 2017) and these activation levels correlate with disease progression (Murdock et al., 2017; McCombe et al., 2020). Macrophages exhibit a similar trend of upregulation in peripheral immunity and neuroinflammation that promote disease progression (Steinacker et al., 2018). Rusconi et al. (2017) suggested that DCs are reduced in the peripheral blood of ALS patients, and these cells are functionally altered and more likely to skew T cell responses towards a pro-inflammatory phenotype in neurodegenerative diseases (Gonzalez and Pacheco, 2014). Despite the controversy regarding the status of peripheral blood CD4 and CD8 T cells in ALS, our results indicate that peripheral CD4 T cells, especially CD4 T naive cells, and CD8 T cells, may be protective factors for survival in patients with ALS. Besides, patients with Cluster2 had a higher immune enrichment, which also implies that NMRGs may improve survival of ALS patients by regulating peripheral immunity. This immunomodulatory mechanism may be related to the level of infiltration of CD4 naive T cells, CD8 T cells, neutrophils and macrophages. Although our results are not able to elucidate the pattern of regulation of peripheral immunity by NAD+ metabolism but demonstrate the associations between them and the relationship of peripheral immunity with the prognosis of patients. Interestingly, we also observed a significant enrichment of GPCR signaling-related pathways in patients with Cluster4. Aberrant transport of GPCRs signaling-mediated neurotransmitters (Husted et al., 2017) and neurotrophic factors can lead to a variety of neurological and psychological disorders, including ALS (Jeanneteau and Chao, 2006). Therefore, the study of the triadic relationship between ALS- NAD+ metabolic- GPCRs signaling is worth further exploration.

This study systematically describes the importance of NAD+ metabolism-related transcription patterns in whole blood for the assessment of survival, as well as constructing a more accurate signature to predict the prognoses of ALS patients. Furthermore, we have briefly analyzed the association between NAD+ metabolism and immune cell infiltration, providing therapeutic directions for modulating peripheral immunity in ALS. Nevertheless, there are several limitations existed in this study. Although we included as many datasets as possible for rigorous validation, we also acknowledge that further cohorts with larger samples are needed to validate our findings. We are devising a cohort study on fluid biomarkers of ALS and currently have a bank of blood samples from ALS patients in the Chinese population (Xie et al., 2014; Deng et al., 2017) that we hope to validate within it. Meanwhile, part of the NMRGs in the dataset was filtered at the microarray quality control stage, resulting in the inability to perform analysis for all NAD+ metabolic genes. Although the prognostic signature demonstrated a better predictive ability in this study, adjustment and optimization of the signature will be necessary for future studies.

As a highly energy-consuming neurodegenerative disease (Greenwood, 2013; Wills et al., 2014), our study first suggests that NAD+ synthesis pathways in peripheral blood of ALS patients are associated with survival. Hypoactive *de novo* synthesis along with hyper-synthesis of non-de novo pathways demonstrated a relatively poor prognosis and vice versa. Furthermore, an NAD+ metabolic risk signature developed by machine-learning algorithms exhibited promising prognostic value, as well as generated a nomogram to quantify risk assessment. Meanwhile, the clinical classifications were analyzed and compared between various risk statuses. Lastly, the association of prognostic risk signature with immune cell infiltration was also assessed.

## Conclusion

In conclusion, our study provides some valuable insights into the introduction of NAD+ metabolic profiling in peripheral blood for ALS risk prediction and treatment decisions, ultimately facilitating the precise management of patients.

## Data Availability

The datasets presented in this study can be found in online repositories. The names of the repository/repositories and accession number(s) can be found in the article/Supplementary Material.

## Abbreviations

ALS, Amyotrophic lateral sclerosis; ALSFRS, Amyotrophiclateral sclerosis functional rating scale; CIBERSORT, Cell-type Identification By Estimating Relative Subsets Of RNA Transcripts; CSF, Cerebrospinal fluid; DC, Dendritic cell; FVC, Forced vital capacity; GSEA, Gene set enrichment analysis; KEGG, The kyoto encyclopedia of genes and genomes; LASSO, The least absolute shrinkage and selection operator; NAD+, Nicotinamide adenine dinucleotide; NR, Nicotinamide riboside; NMN, Nicotinamide mononucleotide; NES, Normalized enrichment score; NK, Natural killer; NMRGs, NAD+ metabolism-related genes; NPRS, NAD+ metabolism-related prognostic risk score; PARPs, Poly (ADP-ribose) polymerases; PCA, Principal component analysis; ROC, Receptor operating characteristic; SIRTs, Sirtuins; UMAP, Uniform manifold approximation and projection for dimension reduction.
