# Supplementary material for: Circulating NAD+ Metabolism-Derived Genes Unveils Prognostic and Peripheral Immune Infiltration in Amyotrophic Lateral Sclerosis
Source: Front Cell Dev Biol. 2022 Jan 28;10:831273. doi: 10.3389/fcell.2022.831273 (PMC8831892; doi:10.3389/fcell.2022.831273)

**Figure S1.** Comparison of PCA before and after data processing of GSE112676 and GSE112680. (A and B) PCA for GSE112676 (A) before and (B) after data processing. (C and D) PCA for GSE112676 (C) before and (D) after data processing.

**
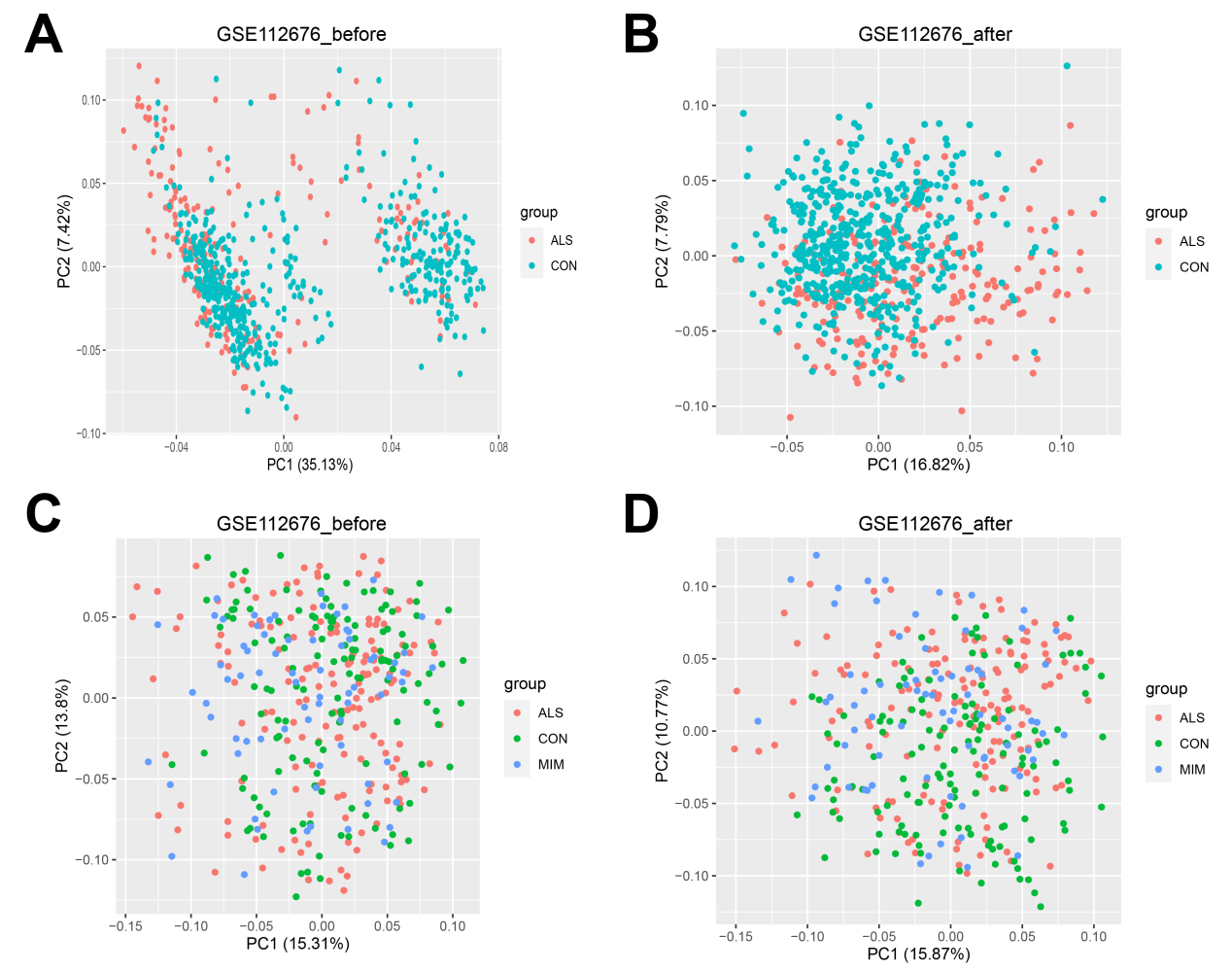
**

**Figure S2.** Consensus clusters by NAD+ metabolic-related genes. (A) Consensus clustering cumulative distribution function for k=2 to 10. (B) Principal component analysis of the four clusters exhibited absolute dissimilarity between the cluster2 and the rest clusters.


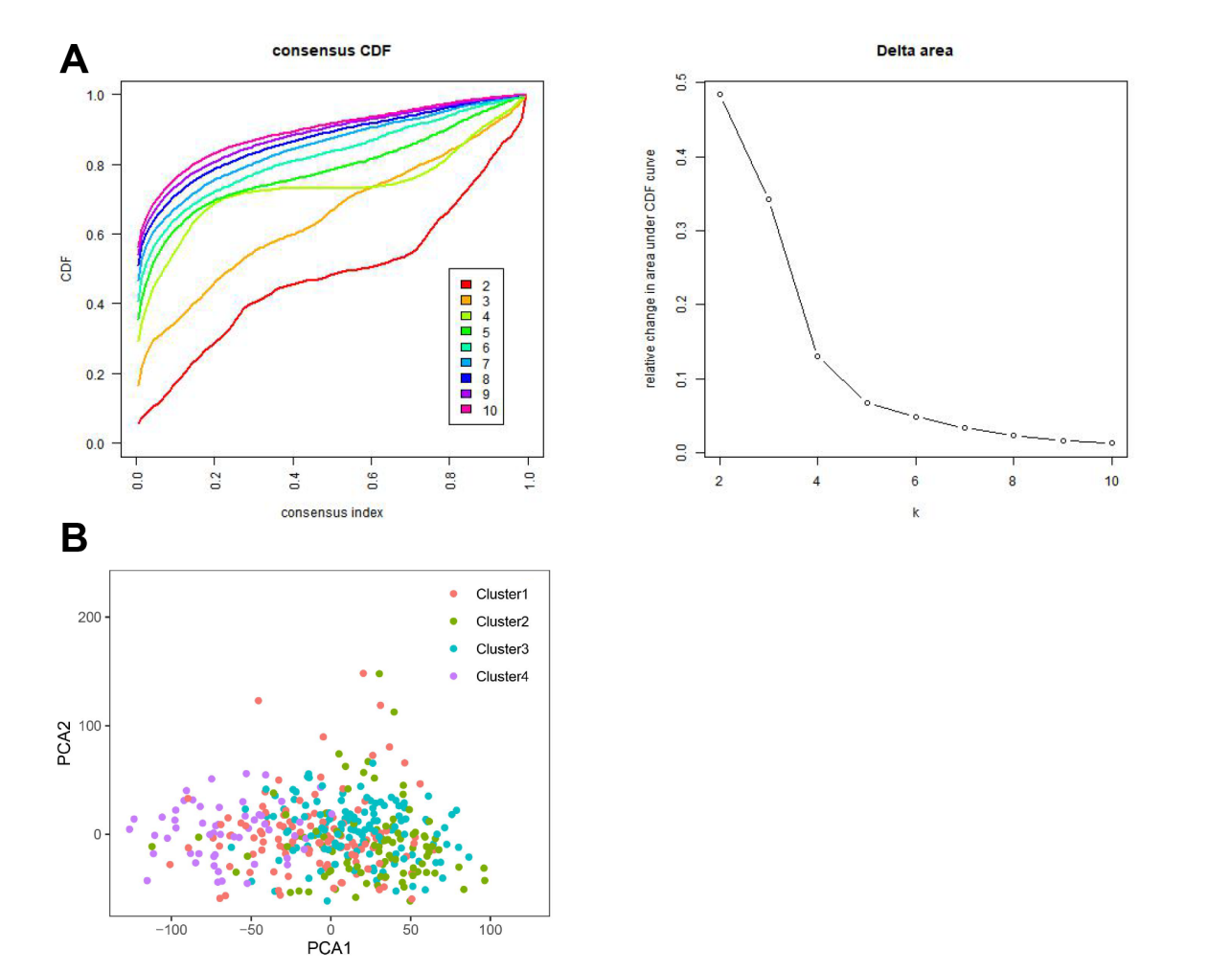


**Figure S3.** LASSO analysis of prognostic NAD+ metabolic-related genes.


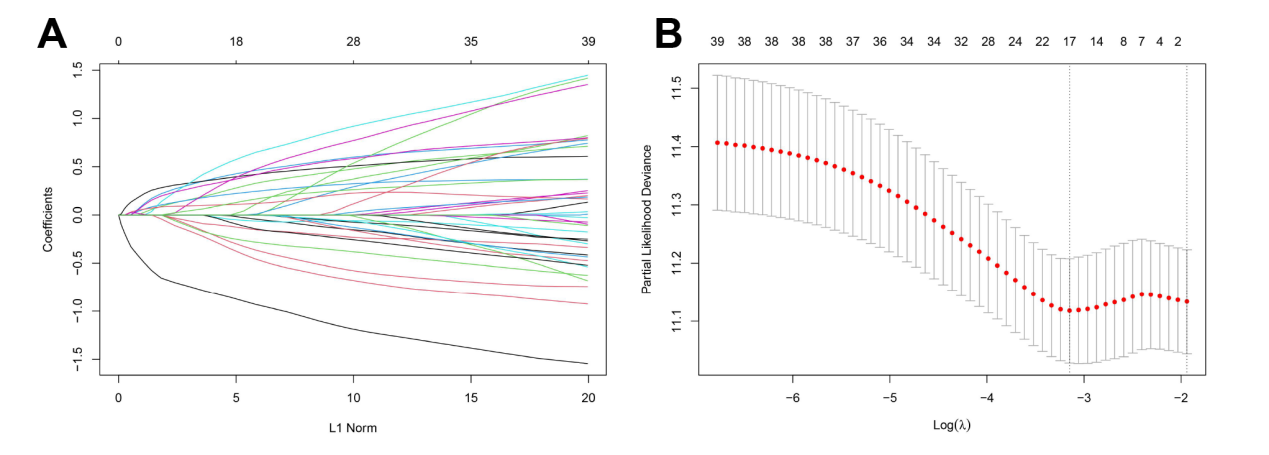


**Figure S4.** The comparison of NPRS among the four subtypes.


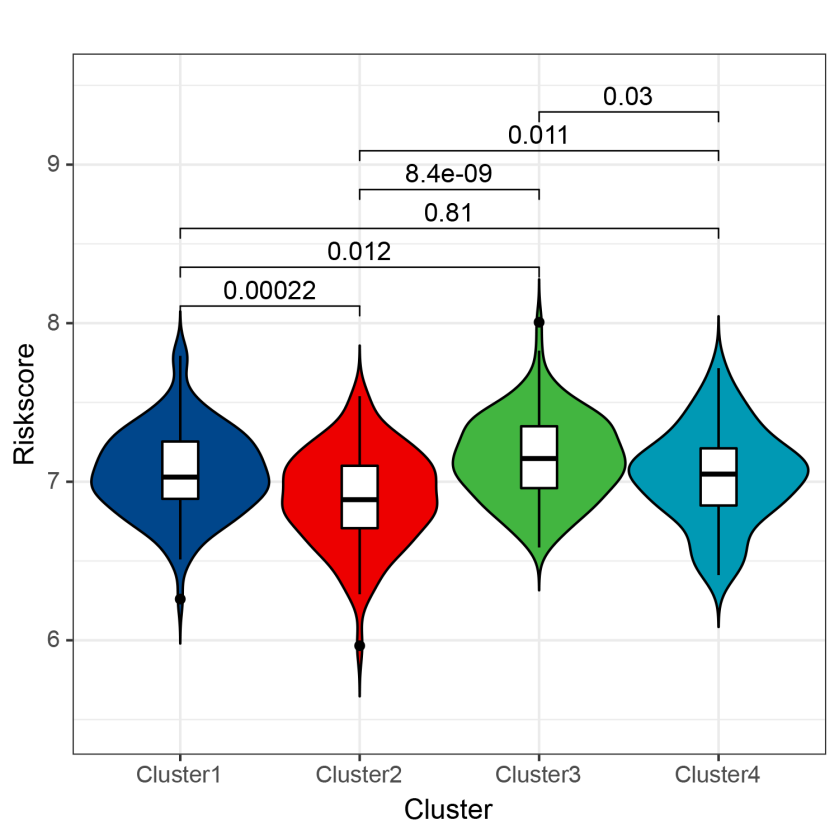


**Figure S5.** The comparison of clinicalpathological parameters between high- and low- NPRS patients.


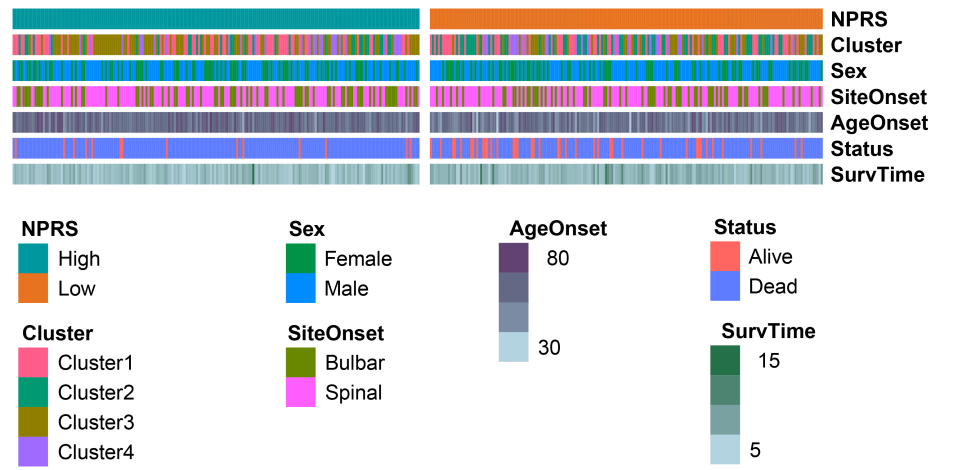


**Figure S6.** Univariate Cox regression analyses in the entire cohort.


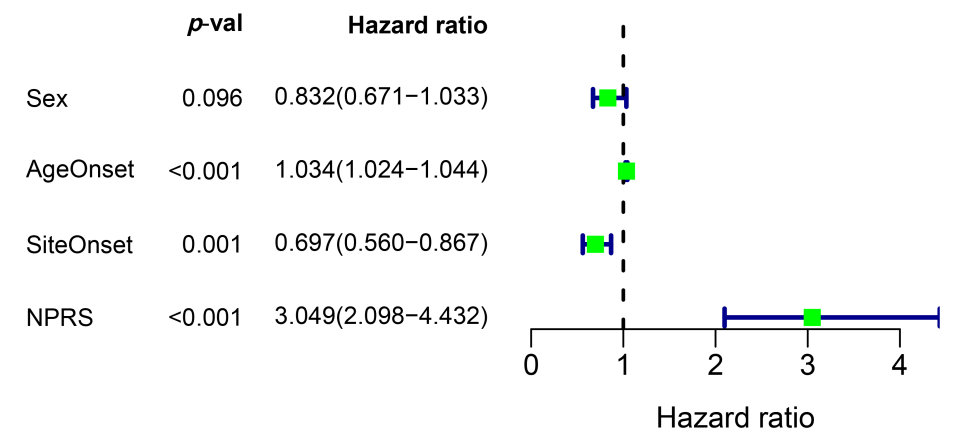

Supplement: Supplementary file 1 [file DataSheet1.docx]
